# Sexually transmitted infections in pregnant women from sub-Saharan Africa

**DOI:** 10.4102/sajid.v36i1.312

**Published:** 2021-12-09

**Authors:** Bongekile Ngobese, Nathlee S. Abbai

**Affiliations:** 1 Department of Clinical Medicine Laboratory, College of Health Sciences, University of KwaZulu-Natal, Durban, South Africa

**Keywords:** sexually transmitted infections, human immunodeficiency virus, pregnant women, health complications, sub-Saharan Africa

## Abstract

**Background:**

Sexually transmitted infections (STIs) are a major health problem in most countries of the world, particularly in developing countries where the resources and technology to diagnose and treat them are limited. Currently, there is limited data on STIs and risk factors for these infections in pregnant women living with human immunodeficiency virus (HIV), especially in sub-Saharan Africa (SSA). This review provides data on the prevalence and risk factors for STIs in pregnant women living with HIV from SSA. This review also describes the association between STIs and HIV on pregnancy and birth outcomes as well as highlights the importance of laboratory-based diagnosis of STIs.

**Method:**

An electronic search of online databases was used to find and collect relevant research articles connected to the prevalence, adverse pregnancy and birth outcomes, health complications and risk factors associated with STIs and HIV in pregnant women from SSA. The search was limited to articles published in English. Relevant studies were identified by searching literature from January 2001 to date. The search yielded 4709 results.

**Results:**

In SSA, STIs are highly prevalent in pregnant women and are widely known to be linked with an increased risk of poor maternal and neonatal outcomes. These infections are often asymptomatic and highly prevalent in pregnant women. The screening of STIs in pregnant women living with HIV can reduce the risk of mother-to-child transmission (MTCT) and screening and treatment for STIs can also prevent adverse perinatal outcomes. It is important to recognise regional and national STI epidemics in order to promote STI prevention and control interventions considering the test and treat approach as opposed to syndromic management.

**Conclusion:**

This review highlights the need to use diagnostic screening methods instead of syndromic STI management in SSA. Moreover, more research into effective prevention and treatment measures for STIs in pregnant women is urgently required.

## Introduction

*Chlamydia trachomatis, Neisseria gonorrhoeae* and *Trichomonas vaginalis* are amongst the most common sexually transmitted infections (STIs) globally,^1,2^ with *T. vaginalis* reported as the most common STI infection in the world.^3,4,5,6^ *Mycoplasma genitalium* is an emerging cause of the genital discharge and has been involved in urogenital infections of men and women worldwide.^7,8^ In many countries, these curable STIs are most common in pregnant women.^1^ However, there are limited reports on the burden of *C. trachomatis, N. gonorrhoeae, T. vaginalis* and *M. genitalium* amongst pregnant women living with HIV in sub-Saharan Africa (SSA).^7,9^ Few countries recommend regular screening for STIs in pregnant women.^10^ However, STI rates vary by country and demographic group, STI rates in pregnant women are higher than in the general population, as young adults typically carry the highest burden of STIs.^11^ These STIs poses a threat to public health, to pregnant women and neonatal health as they may be transmitted to the newborn.^4,12^ The World Health Organization has recommended that all pregnant women be screened for HIV and syphilis during their first antenatal care visit.^3^ Most African countries have adopted WHO recommendations for HIV and syphilis screening at the first antenatal visit, but not all African countries have implemented it yet.^13,14,15^ As a result, these infections are still reported to cause adverse birth outcomes.^16,17^ Recent studies in SSA have shown a high prevalence of curable STIs amongst pregnant women, and therefore the burden of STIs and HIV in SSA remains a significant threat to reproductive health.^18,19,20^ Women infected with STIs can experience a variety of symptoms, including vaginal itching or irritation, vaginal discharge and dysuria, as well as more severe complications such as pelvic inflammatory disease and fallopian tube pathology.^6,21^ Untreated STIs are related with adverse outcomes during pregnancy including stillbirth, preterm labour and delivery and low birth weight.^20,22,23,24^ Furthermore, STIs have been shown to increase the risk of HIV acquisition and mother-to-child transmission (MTCT), and the risk is highest in women with multiple STIs.^2,25,26^

Sub-Saharan Africa bears a heavy HIV burden,^27^ and South Africa has the vast majority of people living with HIV worldwide, with 7.9 million people living with HIV in 2018.^28^ Studies have reported that education, poverty, number of sexual partners, intravaginal practices, STIs history in partners and having sex under the influence of alcohol or drugs are the risk factors of STIs.^29,30,31^ In most cases, STIs are asymptomatic particularly in pregnant women.^9,10,24^ Syndromic management has been implemented in numerous countries particularly in the low- and middle-income countries like South Africa.^3,32,33^ The syndromic approach treats STIs according to the presence of clinical signs and symptoms, rather than by laboratory-based diagnosis.^20,32^ However, the inadequate specificity and sensitivity of this approach contributes to both over and undertreatment and poor antimicrobial stewardship may increase the risk of antibiotic resistance.^1,34^ As a significant percentage of women who have STIs are asymptomatic, they are left untreated, and this may contribute to many adverse birth outcomes.^2,35,36^ Recent research to integrate STI molecular diagnostic tests into antenatal care services has demonstrated that it is appropriate and viable for pregnant women living with HIV.^37^

### Review methodology

An electronic search of the following databases was conducted: PubMed/MEDLINE and Google Scholar. The search terms included prevalence, adverse birth outcomes, epidemiology, health complications, risk factors, pregnant women, STIs, HIV, syphilis and SSA. Boolean terms (AND, OR) were used to separate the keywords, and Medical Subject Heading (MeSH) terms were included during the search. Websites such as WHO and governmental websites were searched for policies and guidelines regarding diagnosis and management of STIs in pregnancy. The search was limited to articles published in English. Relevant studies were identified by searching literature from January 2001 to date. The search yielded 4709 results. Articles were also searched through the ‘Cited by’ search as well as citations included in the reference lists of included articles. The following criteria were used for selection:

### Inclusion criteria

1. Studies reporting the prevalence or incidence of STIs in pregnant women from SSA only.
2. Full articles available in English. Age, study sample size and laboratory method used for the detection of pathogens were not restricted.

### Exclusion criteria

1. Studies reporting the prevalence or incidence of STIs in non-pregnant women, adolescents, female sex workers and men who have sex with men were not considered.
2. Articles not available as full text and in English.
3. Articles that were only available as abstracts but not accessible (required to be purchased).

### Epidemiology of sexually transmitted infections in sub-Saharan Africa

In 2012, the World Health Organization (WHO) estimated that there were 357 million incident cases of the curable STIs worldwide, 143 million cases of *T. vaginalis*, 131 million cases of *C. trachomatis*, 78 million cases of *N. gonorrhoea* and 5.6 million cases of syphilis.^3^ The STIs are a major global public health issue, with an estimated number of 500 million new cases of curable STIs, most of which arise in low- and middle-income countries (LMICs) settings.^38^ Past studies have documented that there are very few population-based STI prevalence studies especially for LMICs.^39,40,41^

In the developing world, the vast majority of curable STIs are reported for the African region. In 2008, the WHO reported that women in the African region had high prevalence estimates for *N. gonorrhoeae, C. trachomatis* and *T. vaginalis* at 2.3%, 2.6% and 20.2 % compared to 2.1%, 2.0% and 2.0% for men, respectively.^27^ Moreover, the WHO had reported that 8.3, 21.1 and 59.7 million new cases of *C. trachomatis, N. gonorrhoeae* and *T. vaginalis* infections occur in SSA, with most new STIs emerging amongst the population aged 15–49 years.^27^ *Mycoplasma genitalium* is an emerging STI and is associated with HIV acquisition, especially in SSA women, but it is not known whether women with *M. genitalium* transmit HIV more easily to male sexual partners.^8^ *Mycoplasma genitalium* has been detected in 5% – 20% of sexually active African adults and is most prevalent amongst adults with HIV infection.^42^ In Africa, *M. genitalium* is not regularly screened and neither effectively treated by WHO syndromic management guidelines for STIs.^43^

Recent modelling analysis identified SSA as the region with the highest incidence and prevalence of STIs. Although data on the distribution of STIs in countries in SSA are widely disseminated (over 100 studies), several countries report on the prevalence of syndromes rather than on individual pathogens.^44^ Amongst these curable STIs, *C. trachomatis, N. gonorrhoeae* and *T. vaginalis* are major public health problems amongst women, but the burden of infection in SSA is not well documented.^44,45^ Moreover, in a recent study by Hoffman et al.,^35^ it was found that there is limited data on the burden of STIs in SSA and linkage to care for these infections. Previous studies have reported that one in four women in South Africa is infected with at least one bacterial STI.^46^

### Sexually transmitted infections in pregnant populations

The prevalence of curable STIs in pregnant women in LMICs is significant in all areas, indicating a high burden of infections at the population level.^11,30,47^ In SSA, where antenatal and healthcare are limited and maternal and infant morbidity is high, it is important to understand the burden of STIs and its impact on female reproductive health, maternal health and neonatal consequences.^48,49^ Most recently, in developing countries because of STIs, the WHO reported a stillbirth rate of 25.6 per 1000 births in 2009 and a neonatal mortality rate of 28 per 1000 births in 2015 compared to 2.4 and 2.0 in developed countries, respectively.^50^ Recently published data showed that in numerous regions of the world antenatal screening for STIs is not regularly practiced, particularly in LMICs. However, regular screening may be beneficial for pregnant women living with HIV and their infants.^25,51^ Moreover, Schonfeld et al.^52^ reported that there is limited data on the prevalence and association of STIs in pregnant women by HIV status in LMICs, where syndromic STI management is routine. Furthermore, Joseph et al.^18^ reported that in LMICs, the prevalence of curable STIs such as *N. gonorrhoeae, C. trachomatis* or *T. vaginalis* can be as high as 25% in pregnant women.^18^

There is a lack of data on the prevalence and associated risk factors of *C. trachomatis, N. gonorrhoeae* and *T. vaginalis* in pregnant women, especially in SSA, which bears a disproportionate burden of HIV.^9^ The approximate average prevalence of these conditions in specific African countries amongst antenatal participants is 5.1% for *N. gonorrhoeae*, 11.3% for *C. trachomatis* and 15.2% for *T. vaginalis*.^53^ In Africa, there is currently no accurate approximation available for incidence of *M. genitalium* infection, but a prevalence range of 6% – 11% amongst women has been reported.^54,55^

High levels of untreated STIs in SSA are linked to high transmission rates of HIV and are thought to have contributed to the high HIV prevalence in the region.^56^ To date, with 25.6 million people living with HIV, SSA remains the most affected country.^52^ The lack of data in SSA demonstrates the urgent need to collect accurate STI prevalence measures in LMICs, where the burden is the highest amongst pregnant women. Furthermore, the WHO Global Health Sector Strategy on STIs 2016–2021 has outlined the priorities and objectives for global STI prevention and control. The first strategic direction is to gather data on the prevalence and incidence of STI amongst representative populations.^3^ In SSA, prevalence estimates for *C. trachomatis* amongst antenatal women ranged from 4.6% to 14.9%.^5,8,45,51,52,53,57,58,59^ The prevalence estimates for *C. trachomatis* amongst antenatal women ranges from 6.5% to 36.8% in South Africa (Table 1).^2,9,18,19,20,22,30,36,37,60^

**TABLE 1:** Summary of prevalence data for sexually transmitted infections in pregnant women from sub-Saharan Africa.

References	Prevalence rates (%)	Country
***C. trachomatis***		
Adachi et al.,^30^	21.3	South Africa
Moodley et al.,^19^	17.8	South Africa
Joseph et al.,^22^	17	South Africa
Morikawa et al.,^37^	29.5	South Africa
Mudau et al.,^9^	36.8	South Africa
Joseph et al.,^18^	20	South Africa
Green et al.,^61^	13	South Africa
Mediana et al.,^36^	26.5	South Africa
Nyemba et al.,^20^	15.3	South Africa
Nyemba et al.,^20^	14.8	South Africa
Smullin et al.,^60^	6.5	South Africa
Smullin et al.,^60^	12	South Africa
Gadoth et al.,^57^	3.1	Congo
Gadoth et al.,^5^	3.2	Congo
Masha et al.,^45^	14.9	Kenya
Roxby et al.,^8^	4.6	Kenya
Abdelrahim et al.,^53^	4.9	Sudan
Offorjebe et al.,^58^	7.0	Botswana
Wynn et al.,^59^	13.5	Botswana
Schonfeld et al.,^52^	9.8	Ethiopia
Juliana et al.,^51^	4.6	Tanzania
***N. gonorrhoeae***		
Adachi et al.,^30^	7.6	South Africa
Moodley et al.,^19^	6.4	South Africa
Joseph et al.,^22^	0.9	South Africa
Morikawa et al.,^37^	5.6	South Africa
Mudau et al.,^9^	6.9	South Africa
Joseph et al.,^18^	5.8	South Africa
Green et al.,^61^	1.0	South Africa
Nyemba et al.,^20^	0.9	South Africa
Nyemba et al.,^20^	0.7	South Africa
Mediana et al.,^36^	6.3	South Africa
Smullin et al.,^60^	1.1	South Africa
Smullin et al.,^60^	0	South Africa
Gadoth et al.,^57^	1.4	Congo
Gadoth et al.,^5^	1.5	Congo
Masha et al.,^45^	1.0	Kenya
Roxby et al.,^8^	21.1	Kenya
Abdelrahim et al.,^53^	0	Sudan
Schonfeld et al.,^52^	4.3	Ethiopia
Offorjebe et al.,^58^	1.0	Botswana
Wynn et al.,^59^	1.3	Botswana
Asmah et al.,^21^	2.0	Ghana
Juliana et al.,^51^	0	Tanzania
***T. vaginalis***		
Moodley et al.,^19^	15.3	South Africa
Joseph et al.,^22^	10.0	South Africa
Morikawa et al.,^37^	20.2	South Africa
Mudau et al.,^9^	23.9	South Africa
Prince et al.,^6^	20.0	South Africa
Joseph et al.,^18^	15.0	South Africa
Dessai et al.,^64^	10.3	South Africa
Green et al.,^61^	10	South Africa
Mabaso et al.,^65^	59.6	South Africa
Mediana et al.,^36^	11.6	South Africa
Nyemba et al.,^20^	10.3	South Africa
Nyemba et al.,^20^	5.9	South Africa
Smullin et al.,^60^	8.7	South Africa
Smullin et al.,^60^	4.8	South Africa
Gadoth et al.,^57^	14.6	Congo
Gadoth et al.,^5^	14.0	Congo
Masha et al.,^45^	7.4	Kenya
Masha et al.,^62^	6.5	Kenya
Roxby et al.,^8^	18.6	Kenya
Oyeyemi et al.,^4^	18.7	Nigeria
Abdelrahim et al.,^53^	7.8	Sudan
Schonfeld et al.,^52^	5.3	Ethiopia
Offorjebe et al.,^58^	6.0	Botswana
Wynn et al.,^59^	5.0	Botswana
Asmah et al.,^21^	20.2	Ghana
Juliana et al.,^51^	7.7	Tanzania
***M. genitalium***		
Smullin et al.,^60^	24	South Africa
Smullin et al.,^60^	12	South Africa
Roxby et al.,^8^	21.4	Kenya
Juliana et al.,^51^	2.1	Tanzania

Studies conducted in SSA amongst antenatal women reported prevalence estimates for *N. gonorrhoeae* ranging from 0% to 21.1%.^5,8,11,21,45,51,52,53,57,58,60^ In South Africa amongst women attending antenatal clinic, the prevalence estimates for *N. gonorrhoeae* range from 0% to 6.9% (Table 1).^2,9,18,19,20,22,30,36,37,60^ Studies conducted in SSA in antenatal women reported prevalence rates of *T. vaginalis* ranging from 5% to 18.7%.^4, 5,8,21,45,51,53,57,58,59,62,63^ The prevalence of *T. vaginalis* amongst antenatal women in South Africa ranges from 4.8% to 59.6% (Table 1).^2,6,9,18,19,20,22,36,37,60,64,65^ The prevalence estimates for *M. genitalium* amongst antenatal women in SSA ranged from 2.1% to 21.4%.^8,51^ In a study conducted by Smullin et al.^60^ amongst HIV-infected and HIV-uninfected pregnant women attending an antenatal clinic in South Africa, prevalence rates of 24% and 12% for *M. genitalium* were reported (Table 1).

Studies have shown that the risk of adverse effects in pregnancy and newborns is increased when coinfections of two or more organisms are present.^47^ Moreover, it has been reported that infection with more than one STI is more prevalent in women living with HIV compared to women living without HIV.^18^ However, the consequences of coinfections are largely uncertain complicating empirical treatment, which serves as the basis for STIs syndromic management.^27^ Most studies have reported that the rate of having multiple infections is much more common in younger women aged 16–19 years than in those aged above 35 years.^66,67^ A study conducted in South Africa amongst pregnant women living with HIV reported the rate of coinfections to be 2.6% for *C. trachomatis* and *N. gonorrhoeae*, 8.6% for *C. trachomatis* and *T. vaginalis*, 0.7% for *N. gonorrhoeae* and *T. vaginalis*, and 1.4% had all three STIs.^22^ Another study by Joseph et al.^18^ reported that in pregnant women living with HIV, 50% of those were coinfected when compared with 16% of women living without HIV who were coinfected (*p* < 0.01).

### Human immunodeficiency virus and sexually transmitted infections

Sub-Saharan Africa continues to be the most affected region^18,27^ with 25.6 million people living with HIV. Several studies have reported that STIs are associated with increased rates of HIV acquisition and transmission (1.5–5.5-fold increased risk).^9,23,44,51,52,68,69,70^ Moreover, studies have shown that people infected with HIV have a higher risk of STIs compared to people without HIV.^9,18,25^ South Africa is at the epicenter of the HIV epidemic, whilst other STIs remain endemic.^71^ Sexually transmitted infections contribute significantly to the burden of health in South Africa and are known as key contributors to the HIV epidemic.In 2015, the prevalence of HIV infection amongst adults in the reproductive age group was estimated to be 19.2% in South Africa.^19,29,32,72^ In South Africa, approximately 7.9 million people of all ages were living with HIV in 2018.^28^ The high prevalence of HIV coupled with STIs particularly in young women has been reported for KwaZulu-Natal (KZN).^73^ However, recent studies have reported that population prevalence and the associations between the presence of STIs and HIV in KZN need further investigation.^71^ Previous studies have shown significantly higher HIV infection rates amongst women diagnosed with at least one STI when compared to women without STIs.^29,30^ Similar results were obtained by Chirenje et al.,^74^ where the burden of STIs was greater amongst women living with HIV compared to women living without HIV (54.7% vs. 37.2%, *p* < 0.05, respectively). Sexually transmitted infections and HIV are thought to share a complex bidirectional relationship.Moreover, STIs may increase the risk of HIV infection and transmission by a variety of mechanisms, including decreased epithelial barrier integrity, chronic inflammation and increased numbers of HIV target cells in the genital tract.^71,75^The STIs and HIV share the same behavioural, socioeconomic and demographic risk factors, including age at first sexual intercourse, inconsistent use of condoms, having multiple sexual partners, female sex, being single, having a partner who has other partners, location and culture.^56^ Evidence indicates that individuals living with HIV, persistent high-risk behaviours increase vulnerability to STIs and HIV infection can increase the likelihood of STI treatment failures.^68,70,71^

The burden of STIs amongst pregnant women living with HIV in South Africa is not well known.^9,22^ Untreated STIs and HIV in pregnant women have severe neonatal consequences.^10^ Recently published evidence indicates that, amongst pregnant women living with HIV, STIs can increase the risk of MTCT of HIV through increased HIV viral load shedding from genital inflammation and leading to increased cervicovaginal shedding of HIV and chorioamnionitis.^8,9,11,37,45,47^ A study by Adachi et al.^25^ showed that in pregnancy, STIs nearly double the risk of MTCT of HIV in utero and intrapartum. Infections with *C. trachomatis, N. gonorrhoeae* and *T. vaginalis* are important causes of morbidity amongst pregnant women living with HIV and are associated with adverse pregnancy and birth out comes particularly *C. trachomatis* and *N. gonorrhoeae* infections have been shown to increase the risk of MTCT of HIV infection.^36^ These findings are consistent with the previous study in Botswana, which reported that the prevalence of STIs was high, especially amongst women with HIV infection and coinfection with HIV and *C. trachomatis*/*N. gonorrhoeae* was associated with an increased risk of MTCT of HIV (1.5-fold increased risk [odds ratio], 1.47; 95% confidence interval, 0.9–2.3).^59^ Furthermore, *M. genitalium* is an emerging STI and despite strong evidence that it increases the risk of HIV acquisition and transmission, few studies have evaluated the prevalence and incidence of *M. genitalium* infection in pregnant women living with HIV.^60^ Similarly, Roxby et al.^8^ reported that according to their understanding, the significant effect of *M. genitalium* in MTCT of HIV has not been studied. The STIs increase the risk of HIV acquisition in women living without HIV and high viral load can drastically increase the risk of vertical HIV transmission.^76^ Pregnant women living with HIV infection are at risk of vertical and sexual transmission of HIV and failure to diagnose and treat them for STIs may increase the risk of onward vertical and sexual transmission of HIV.^6,18^

In a study by Adachi et al.,^30^ the rate of HIV MTCT was higher amongst infants born to mothers infected with *C. trachomatis* (10.7%) or both *C. trachomatis* and *N. gonorrhoeae* (14.3%) compared to those born to uninfected mothers (8.1%). They further reported that in utero HIV MTCT was the highest amongst mothers coinfected with *C. trachomatis* and *N. gonorrhoeae* (8.6%) or mothers who had *C. trachomatis* alone (7.5%).^30^ A previous study conducted in Tanzanian women living with HIV reported that coinfection with *N. gonorrhoeae* was associated with a 5.5-fold increased risk of intrauterine HIV transmission.^77^ Trichomoniasis has also been reported to increase the risk of HIV infection by at least by twofold increasing HIV viral shedding up by fourfold.^45,78^ Similarly, a study by Bristow et al.^79^ reported that *T. vaginalis* infection has been associated with a more than 2.7-fold increase in the risk of HIV acquisition, a 1.3-fold increase in preterm labour and a 4.7-fold increase in pelvic inflammatory disease. The sub-study of the National Institute of Child Health and Human Development (NICHD) HIV Prevention Trials Network (HPTN) that was conducted in four countries reported that 35 (42.7%) of HIV-infected infants were born to women with at least one STI, whereas maternal STI rates amongst HIV-uninfected infants was 29.1%. Furthermore, as compared to those without STIs, women with two STIs had more than 3.4-fold to transmit HIV to their infants.^25^ Numerous studies have been conducted on the physiological changes during pregnancy and postpartum phases that increase the risk of HIV and factors that increase risky sex during pregnancy.^80^ The correct management of STIs is urgently required and will reduce the risk of acquiring HIV; more importantly, screening of pregnant women at risk for these infections could also aid in reducing MTCT of both HIV infection and STIs.^75,81^

### Risk factors associated with sexually transmitted infections and human immunodeficiency virus

There is a lack of data on the prevalence of *C. trachomatis, N. gonorrhoeae, T. vaginalis* and *M. genitalium* amongst pregnant women and their related risk factors: mainly in SSA, which bears a significant burden of HIV.^7,9,40^ The infection rates are much higher amongst pregnant women than in the general population. In South Africa, sexual behaviour is not well known in pregnant women infected with HIV.^22^ Similarly, recent studies have reported that data on the prevalence of curable STIs and their risk factors in pregnant women is lacking.^71^ Several studies have reported that risk factors associated with STIs include younger age, condomless sex, being single or unmarried, having more than one sexual partner, unemployment, low level education attainment, frequent alcohol use, frequent tobacco use, illegal substance use and a history of previous STIs.^29,30,31,60,67,73,82,83,84^ Furthermore, a recent study had reported that having oral, anal or vaginal intercourse having multiple partners, having anonymous partners, using alcohol or drugs and not wearing condoms increases the risk for HIV and other STIs.^85^ Several studies have reported that the prevalence of STI in pregnant women younger than 25 years of age is significantly higher when compared to pregnant women aged 25–35 years, but the risk in pregnant women > 35 years of age is declining.^9,25,41,51,86^ Similarly, numerous studies have shown that estimates of curable STIs are higher in younger age groups, especially in younger females, which is the same group with the highest incidence of HIV, thus the importance of enhanced STI surveillance and investment in targeted STI control programmes for younger populations, especially women.^70,71,87^ Early sexual debut and having a higher number of sexual partners have been reported to be strongly associated with curable STIs.^73,83,88,89^ Moreover, studies have reported that intravaginal cleansing is a risk factor that is associated with acquiring STIs.^35^

In resource-poor countries, education and poverty are key STI determinants, and this is because inequality exists, and young, rural, poor and less-educated women may have less access to antenatal clinic (ANC) services.^52^ Higher prevalence of STIs amongst women living with HIV presenting late for their first ANC consultation has been reported. However, there are numerous biological, immunological and behavioural factors that contribute to this increased risk (5.5 increased risk).^9^ It has been reported that women living with HIV not on antiretroviral therapy (ART) have a higher frequency of persistent STIs.^36^ A study from KZN, South Africa, showed that alcohol use by women living with HIV was associated with prior STI treatment and increased numbers of sex partners,^90^ whilst a study conducted in pregnant women in Mpumalanga, South Africa, showed that HIV infection, physical partner violence in the past 6 months, and psychological distress were associated with STI.^91^ Given the increased risk of horizontal and vertical transmission of HIV associated with STIs (1.5 fold increased risk), it is urgently important to consider the sexual and behavioural risk factors associated with STIs during pregnancy.^22^

### Asymptomatic sexually transmitted infections

Many studies have reported that STIs are frequently asymptomatic and mostly affect women.^9,10,52,88^ *C. trachomatis, N. gonorrhoeae, T. vaginalis* and *M. genitalium* may cause asymptomatic disease and are highly prevalent in pregnant women (~50%).^24,38,59^ The high prevalence of asymptomatic infections in pregnancy is because of physiological changes, such as changes in vaginal discharge and urinary habits, which may mask the signs and symptoms of true infection.^37^ The high prevalence of asymptomatic STIs in pregnant women remains undiagnosed and untreated for a long period, thereby leading to serious complications.^8,31,44,67^ Untreated STIs have been linked to complications such as adverse pregnancy and perinatal outcomes, chronic pelvic illnesses and increased risk of HIV acquisition (1.52-fold increased risk).^87,92^ Furthermore, complications from these asymptomatic STIs can result in severe morbidity and mortality.^40^ Sexually transmitted infections management is a significant public health issue because of the combination of high STI prevalence, high proportion of asymptomatic infections and adverse perinatal and neonatal outcomes.^27^ Joseph et al.^18^ reported that most pregnant women who tested positive for at least one STI were asymptomatic. A study by Masha et al.^45^ reported that 45.2% of their study women with curable STIs were asymptomatic. A study conducted in Cape Town, South Africa, amongst pregnant women reported that most women infected with *M. genitalium* were asymptomatic (77%).^60^ Similarly, a study conducted in Soshanguve Township, South Africa, amongst HIV-infected pregnant women reported that high rates of *T. vaginalis* were asymptomatic.^6^ A recent study from Brazil had reported that 50% – 80% of women infected with *C. trachomatis* do not develop symptoms, and these women are considered silent reservoirs of the pathogen and continue to transmit it sexually.^93^ Hoffman et al.^35^ reported that amongst asymptomatic women, 49% were diagnosed with an STI but remained untreated under the syndromic approach. In a previous study that was conducted in Papua New Guinea, it was reported that more than half of the study women (53.6 %) had any one of the STIs and 71.6% were asymptomatic.^86^ As most curable STIs are asymptomatic screening is vital for early detection and transmission prevention.

### Untreated sexually transmitted infections are associated with adverse pregnancy and neonatal outcomes

Sexually transmitted infections have been reported to be associated with pelvic inflammatory disease (PID), adverse pregnancy outcomes, cervical cancer, infertility and multiple reproductive tract sequelae.^29,38,40,44,66,67,94,95^ During pregnancy, untreated STIs may lead to a variety of complications including intrauterine death (spontaneous abortion), preterm delivery, intrauterine growth restriction, ectopic pregnancy, postpartum sepsis, congenital infection, stillbirth and low birth weight, miscarriage, neonatal conjunctivitis, neonatal pneumonia, premature rupture of membranes and chorioamnionitis. In men, subsequent subfertility may arise.^9,22,23,24,30,31,41,47,51,52,53^ Sexually transmitted infections can be transmitted vertically to newborns during passage through the birth canal.^36,45,88^ Regular antenatal screening for STIs is not routine practice in many regions of the world, especially in LMICs, despite the high burden and risk to maternal and infant health posed by STIs, and recently published evidence indicates that STIs in pregnant women may increase the risk of MTCT of HIV.^9,22,25,74,81^ Regardless of these complications, STIs are a significant cause of mother and child mortality and morbidity particularly in adolescence and during pregnancy.^11,37,56^ Similarly, it has been recently reported that about 1 million babies are stillborn every year in Africa, and another 1 million babies die in their first month of life.^52^ It has been reported that frequent spontaneous abortions and neonatal deaths are common because of the lack of human resources and adequate healthcare infrastructure for premature newborns.^93^ Studies have shown that when coinfections with two or more microorganisms are present, the risk of adverse effects in pregnancy and neonates is increased.^18^ It has been reported that worldwide, up to 4000 newborn babies become blind every year because of eye infections attributable to untreated maternal *C. trachomatis* and *N. gonorrhoeae* infections.^10^ Chlamydial infections are a significant cause of neonatal pneumonia as 5% – 30% of infants born to mothers with *C. trachomatis* infection can acquire this condition.^45,59,96^ About 50% of maternal *C. trachomatis* and *N. gonorrhoeae* infections are transmitted to the neonate during birth, which can cause eye and lower respiratory tract infections.^81^ However, it has been reported that the mechanism by which chlamydial infection may lead to adverse outcomes in pregnancy is not well understood.^47^

In a study conducted on 1373 pregnant women living with HIV, it was shown that approximately 30% of the cohort had a high prevalence of both *C. trachomatis* and *N. gonorrhoeae*, and these infections were associated with infant low birth weights (42.9% vs. 16.9%, *p* = 0.001) and preterm births (28.6% vs. 10.2%, *p* = 0.008) in women with and without these STIs, respectively.^30^ Earlier studies have estimated that approximately 50% – 70% of infants born to mothers with untreated genital chlamydial infection will become infected with 30% – 50% developing conjunctivitis and 10% – 20% developing pneumonia.^25,47^ Similarly, Mullick et al.^97^ demonstrated a higher incidence of low birth weight and preterm delivery in pregnant women infected with *T. vaginalis*. In a recent study by Fuchs et al.,^84^ it was reported that adolescents diagnosed with *T. vaginalis* during pregnancy had more than a twofold increased likelihood of chorioamnionitis compared to those not diagnosed with *T. vaginalis*.The *C. trachomatis* infection in the third trimester of pregnancy was associated with more than a twofold increased likelihood of preterm birth when compared to participants without *C. trachomatis* during pregnancy.^84^ A 2015 meta-analysis of *M. genitalium* infection in pregnant women reported an approximately twofold increase in the odds of preterm delivery or spontaneous abortion in women with this STI.^98^ Furthermore, a recent study conducted by Smullin et al.^60^ in South Africa reported an association between *M. genitalium* and adverse pregnancy outcomes in HIV-infected pregnant women. Given that these four major STIs are curable infections, most pregnancy and neonatal complications can be prevented with antenatal screening programmes that accurately identify and treat infected women. It has been mentioned that most of these sequelae can be prevented if STI testing and treatment are implemented.^73^ Similarly, in a recent study by Lawson,^85^ it was reported that the increased prevalence of STIs in individuals living with HIV indicates the significance of susceptibility and infectivity cofactors in HIV acquisition and transmission and also highlights the importance of early diagnosis and treatment of STIs to control the acquisition and transmission of HIV.

### Importance of laboratory diagnosis for sexually transmitted infections

Traditionally, in SSA and other constrained resource settings in Africa and Asia, STIs are managed syndromically instead of by laboratory diagnosis of specific infections that remains endorsed by the WHO.^3^ Accurate nucleic acid-based diagnostic tests such as multiplex polymerase chain reaction (PCR) assays are now commonly used in high-income countries but are largely unavailable in low- and middle-income countries (LMICs), where the highest prevalence of these STIs and their related adverse health effects occurs.^38,66^ Nucleic acid amplification tests (NAATs) are the diagnostic standard of care tests because of their wide availability and high sensitivity (98% – 100%) and specificity (95%).^11,47,99^ However, less sensitive diagnostic methods such as direct immunofluorescence (IF) and enzyme-linked immunoassays (ELISA) remain in use in lower resourced settings.^94^ Several studies have shown that without the implementation of diagnostic tests, a significant improvement in STI management in resource-poor settings appears to be challenging.^46^ Rapid diagnostic tests for HIV and syphilis are now available in antenatal clinics in many low resourced settings, but simple, easy to use, affordable, highly sensitive and specific rapid diagnostic tests for *C. trachomatis, T. vaginalis, M. genitalium*, and *N. gonorrhoeae* are not yet widely available.^83^ Numerous NAATs, including non-invasive samples taken by the patient (urine) that detect the same amount of disease as samples taken by experts (genital swabs), have been designed for use with several sample types. Self-collected samples may be urine or rectal swabs for both males and females or vaginal swabs in females.^79,100^ Recent developments in technology have introduced the possibility that antenatal testing for *C. trachomatis, N. gonorrhoeae* and *T. vaginalis* infections will become more accessible with highly sensitive, easy to use and rapid tests.^59^ For example, several NAAT platforms are in development or newly available for diagnosis of STIs at the point of care.^59^ Point-of-care (POC) testing, particularly the use of increasingly available NAAT devices, would also enable the testing of asymptomatic women.^74^ The inability to diagnose curable STIs has been a major obstacle to their control, as many cases remain undetected and thus untreated, with the potential for further transmission.^38^ Inability to diagnose asymptomatic infections further limits the availability of epidemiological data that can help to better understand the burden of STIs.^40^ The PCR techniques allow the amplification of DNA from minimal amounts of sample DNA with high sensitivity and specificity although with higher cost.^93^ Diagnostics innovations may allow for the expansion of STI testing and improve management, especially in low resource settings. As a high proportion of women are often asymptomatic, such an approach would have a significant impact on STI control than the existing approach, syndromic management.^74^

Multiplex quantitative PCR has gained popularity over conventional microbiological culture methods.^31^ The advantages of these tests include ease of use and simultaneously detects multiple STIs, and samples can be patient collected, thus reducing storage and shipping costs.^42,59,79^ Rapid testing also has the potential to significantly reduce the time between testing, results, and treatment, which may increase the chance of STI treatment and consequently improve rate of cure.^10,81^ Despite these benefits, diagnostic tests have its limitations; it requires a large financial budget (costly) and human resources and infrastructure investment.^37^ In a study by Cabeza et al.,^101^ it was suggested that the implementation of POC diagnostic screening for STIs into ANC services would increase the detection of women with curable STIs who would have been missed if programmes depend solely on the syndromic screening algorithms. The lack of diagnostic tests for curable STIs during pregnancy may represent a missed opportunity to decrease the burden of infection and poor maternal outcomes.^59^ Diagnostic tests will not only help to diagnose and treat of the most prevalent STIs in women and the general population but can also minimise treatment with unnecessary antibiotics.^46^ To improve screening for and treatment of STIs in pregnancy, advanced diagnostics should be used to prevent maternal morbidity, adverse pregnancy outcomes and transmission to neonate.^81^

## Recommendations

Numerous studies have been carried out on STIs in SSA amongst people attending healthcare facilities or in key groups, such as sex workers and men who have sex with men. However, limited research exists on pregnant women living with HIV and adolescent women who are also at increased risk of getting STIs especially in South Africa where the global prevalence of HIV/STIs is highest. Therefore, research addressing STIs is urgently needed especially in pregnant women living with HIV. The screening of STIs in pregnant women living with HIV can reduce the risk of MTCT, and screening and treatment for STIs can also prevent adverse perinatal outcomes. As in most SSA regions, screening of HIV and syphilis is routine using POC test in antenatal clinics; therefore, pregnant women should also be screened for other STIs. Programmes and campaigns that speak about the importance of STIs screening and antenatal services that are offered for free by government hospitals should be implemented particularly in deep rural and poor communities. Furthermore, the priorities and strategies for global STI prevention and control have been outlined in the WHO Global Health Sector Plan on STIs 2016–2021. The first strategic approach is to gather data on the prevalence and incidence of STIs through representative populations. It is important to recognise regional and national STI epidemics to promote, finance, prepare and enforce STI prevention and control interventions. It also advises LMICs to switch from the STI syndromic method to aetiological monitoring of STIs and to conduct systematic screening in the main population groups at highest risk for STIs, including adolescents.
